# Evaluation of the Information on Dutch Dental Practice Websites Regarding the Treatment of (Frail) Elderly Patients

**DOI:** 10.1016/j.identj.2024.10.023

**Published:** 2024-12-16

**Authors:** Johanna Margaretha Kroese, Brigitta Yue Zhi Li, Samuel Julian The, Jan Joseph Mathieu Bruers

**Affiliations:** a Department of Oral Medicine, Academic Centre for Dentistry Amsterdam, Amsterdam, the Netherlands; b Department of Oral Public Health, Academic Centre for Dentistry Amsterdam, Amsterdam, the Netherlands; c Department of Research and Information, Royal Dutch Dental Association (KNMT), Utrecht, the Netherlands

**Keywords:** Oral healthcare, Frail elderly, Gerodontology, Online information, Websites, Content evaluation

## Abstract

The number of elderly people worldwide and their need for dental care increases in a yearly basis. A dental practice website can be a valuable source of information to these patients that may need comprehensive or adjusted care. Therefore, this study aimed to evaluate the information on Dutch general dental practice websites on topics relevant to the treatment of (frail) elderly patients. Websites were selected from (1) a random sample of 217 Dutch general dental practices (sample-group) and (2) dental practices of 130 members of the Dutch Association for Gerodontology (gero-group), and screened for items in 4 categories: website functionality, contact details and accessibility, composition of the dental team, and specific information for elderly patients. Of the selected websites, 196 in the sample-group and 54 in the gero-group were eligible for data collection. Most websites mentioned opening hours, a phone number, and the composition of the dental team. Other information was overall scarce, especially regarding (frail) elderly patients. The gero-group significantly more often mentioned elderly as a specific target patient group (11,1% vs 2,6%), but numbers were overall low. Websites in the gero-group were hardly more informative or adjusted to elderly than in the sample-group; wheelchair accessibility was the only feature mentioned significantly more often (37.0% vs 17.3%). Only one website in the sample-group and 2 websites in the gero-group mentioned the possibility of home visits. In conclusion, dental practice websites lack information in general, and information relevant to elderly patients in particular, and there is room for improvement in website functionality. Additionally, general dental practices seem insufficiently equipped for the treatment of (frail) elderly patients, highlighting the need for improvement of oral healthcare for this specific patient group. This study addresses the insufficiencies in the provision of information on dental practice websites, allowing for targeted future studies towards improvement.

## Introduction

Worldwide, the number of elderly people increases. In the Netherlands, it is expected that 25% of the population will be 65 years or older by 2040.^1^ Increasing age comes with an increased prevalence of chronic diseases, which in turn is associated with a higher burden of oral health problems.^2^ Nowadays, elderly people retain their natural teeth to an older age,^3^ further increasing the need for dental care. The majority of Dutch people regularly visit the dentist for routine check-ups. However, the frequency of dental care utilisation decreases with age, especially in the frail elderly,^4^ due to barriers such as chronic diseases and decreased mobility.^2^ Further, there is a trend of elderly people living at home longer, increasing their reliance on general dental practices. General dentists may experience barriers for the treatment of (frail) elderly patients due to insufficient knowledge, the complexity of the required care, or inadequate reimbursement.^5^ In the Netherlands, dental services are primarily provided by private practices, and all those patients aged 18 and over typically rely on private insurance or out-of-pocket payments for dental treatment, while the standardised fees for dental treatments often insufficiently cover the additional time and resources required to care for frail elderly patients.

In the Netherlands, several initiatives exist to improve oral healthcare for (frail) elderly patients, including a dedicated practice guide on this topic aimed at general dentists and other oral healthcare professionals, published by the Royal Dutch Dental Association.^6^ Further, it is encouraged to offer a home visit in case a patient is not able to visit the dental practice, and a clinical practice guideline (CPG) on this topic was published by the Dutch organisation for the development of CPGs (*Kennisinstituut Mondzorg;* KIMO) to support general dentists.

The elderly are increasingly using digital technology, with a majority being connected to the internet, and generally have adequate digital literacy.^7^ For patients in general, but especially for patient groups that may need comprehensive or adjusted care such as the (frail) elderly, a dental practice website can be a valuable source of information on several topics, for example, accessibility of the dental practice and the possibility of home visits. In 2006, only 17% of the Dutch dental practices had a website. This number increased considerably, to 87% in 2013, as a result of the social trend that dental practices were encouraged to be transparent about their rates and various other aspects of their care provision, for example by publishing them on a website.^8^ The question is to what extent dental practices nowadays actually use their website to inform patients about their healthcare offering, in particular the (frail) elderly among them, and whether websites have accessibility features to enable frail elderly patients to access the information. After all, KIMO suggests that information on dental practice websites about the possibility of home visits is an indicator for implementation of their CPG on this topic.

The aim of this study was therefore to evaluate the accessibility features of, and the information on general dental practice websites on topics relevant to the treatment of (frail) elderly patients, as determined by the practice guide of the Royal Dutch Dental Association on this topic. It was hypothesised that websites of practices owned by or employing a dentist member of the Dutch Association for Gerodontology provide more relevant information. Because home visits, although encouraged by authorities, are not (yet) a regular part of Dutch oral healthcare, overall a low number of websites mentioning the possibility of a home visit was expected.

## Material and methods

### Study design

The present cross-sectional registration study was carried out in April 2023, by using publicly available information on dental practice websites. Prior to the study, the protocol was approved by the Internal Review Board Committee of the Academic Centre for Dentistry Amsterdam (ACTA), document number 2023-26664.

### Data collection

#### Sample

Dental practices and corresponding websites were selected in 2 groups: (1) a random sample of 217 general dental practices with registered addresses within the Netherlands, obtained from the national database of dentists maintained by the Royal Dutch Dental Association (sample-group), and (2) the dental practices owned by or employing the 130 members of the Dutch Association for Gerodontology as published on their website (gero-group). Before selecting the sample for group [1], all practices from group [2] were excluded from the database to prevent duplicates. Since the research aim concerns general dental practices, practices were excluded when housed in a hospital or nursing home, not having a physical practice (e.g. organisations that solely provide outpatient care through home visits), solely providing emergency dental care, or being a referral clinic. Additionally, dental practices that were permanently closed or did not have a website were excluded.

#### Research instrument

A list of items relevant to elderly living at home and seeking oral healthcare was composed, based on the practice guide of the Royal Dutch Dental Association.^6^ This practice guide contains several advices concerning oral healthcare for frail elderly patients, for practice owners to consider. The list of items was reviewed, complemented and discussed until consent was reached by a geriatric dentist with experience in the treatment of frail elderly patients (JMK), a specialist on information and research working at the Royal Dutch Dental Association (JJMB) and 2 dental students at ACTA (BYZL and SJT). This resulted in adding items on website functionality, mentioning of the elderly as a specific patient group, and mentioning of relevant CPGs and websites, as a possible indication of the dental practice being aware of the specific needs of the elderly patient group.

The list of items was converted to a registration form in Qualtrics, a web-based software that allows data collection through a custom survey. The final list consisted of several dozen items spread over 4 categories, viz. website functionality, contact details and accessibility, composition of the dental team, and specific information for elderly patients. Appendix Table 1 provides an overview of all items on the registration form.

#### Data registration

The actual registration of information found on all included websites was performed by 2 dental students (BYZL and SJT) of ACTA using the registration form in Qualtrics. First, a small sample was screened by both students and discussed to calibrate for data registration. Thereafter, both students screened each half of the included websites. In case of doubt, websites were discussed together until consensus was reached. Finally, one student checked all registration forms on possible missing data.

### Statistical analysis

Data was exported from Qualtrics and statistical analyses were performed using IBM SPSS Statistics version 28.0 (IBM, New York, NY, USA). Results are reported using descriptive statistics. Differences between the sample-group and gero-group were tested using the Chi-square test, or Kruskal-Wallis test when appropriate. P-values <0.05 were considered statistically significant.

## Results

### Research group

Removing duplicates (sample-group n = 3; gero-group n = 23) and excluding websites based on the exclusion criteria resulted in a final total of 250 websites eligible for data collection – 196 in the sample-group, and 54 in the gero-group. With the 250 included websites, this study comprises 5.8% of the total number of approximately 4300 dental practices in the Netherlands.

In the sample-group, most excluded dental practices did not have a website (n = 11). Other reasons for exclusion were being housed in a hospital or nursing home (n = 3), being permanently closed (n = 2), being an emergency clinic (n = 1), and being a referral clinic (n = 1). In the gero-group, 13 practices did not have a website, but being housed in a hospital or nursing home (n = 12), not having a physical practice (n = 14), and being permanently closed (n = 11) were also common reasons for exclusion. Other reasons for exclusion were being a referral clinic (n = 2) and the dentist having passed away (n = 1).

### Website functionality

The majority of websites did not possess any of the possible features to enhance website functionality (Table 1). Approximately one third of websites had a zoom function, search function and/or day and night mode, possibly because of the use of standard website templates that commonly offer these features. However, adjusting the text size – a feature that could possibly be very useful for elderly people with deteriorating vision – was only possible on one website. Overall, no significant differences were found between the groups.

**Table 1:** Features that enhance website functionality per group

	Sample-group (n = 196)		Gero-group (n = 54)	
	N	%	n	%
One or more features present	85	43.4	25	46.3
- *Search function*	*78*	*39.8*	*19*	*35.2*
- *Zoom function*	*45*	*23.0*	*15*	*27.8*
- *Day and night mode*	*45*	*23.0*	*15*	*27.8*
- *Language change*	*12*	*6.1*	*4*	*7.4*
- *Adjustment of text size*	*-*	*-*	*1*	*1.9*
- *Reading text aloud*	*-*	*-*	*-*	*-*

### Contact details, and accessibility

Results on contact details and accessibility are shown in Table 2. Almost every website mentioned opening hours of the dental practice. Dental practices in the sample-group significantly more often included at least one weekday evening in their regular opening hours. No other differences were found between the groups.

**Table 2:** Information on opening hours, contact details, and accessibility mentioned on the website, per group

	Sample-group (n = 196)		Gero-group (n = 54)	
	n	%	n	%
Opening hours mentioned	183	93.4	51	94.4
- *Weekdays*	*183*	*93.4*	*51*	*94.4*
- Weekday evenings*	*65*	*33.2*	*7*	*13.0*
- *Weekend*	*23*	*11.7*	*3*	*5.6*
- *Weekend evenings*	*1*	*0.5*	*-*	*-*
- *Appointment outside regular opening hours possible on request*	*7*	*3.6*	*2*	*3.7*
Phone number mentioned	196	100.0	54	100.0
- *Hours mentioned during which the dental practice can be reached by phone*	*117*	*59.7*	*36*	*66.7*
- *Emergency phone number mentioned*	*181*	*92.3*	*51*	*94.4*
- *Hours mentioned during which the emergency phone number can be reached*	*166*	*84.7*	*48*	*88.9*
- *Emergency dental care provided at the same location as the general dental care*	*2*	*1.0*	*-*	*-*
Appointment reminders	48	24.5	11	19.6
- *E-mail*	*34*	*17.3*	*9*	*16.7*
- *SMS*	*19*	*9.7*	*7*	*13.0*
- *Mail*	*2*	*1.0*	*1*	*1.9*
- *Phone*	*2*	*1.0*	*-*	*-*
Regular parking spaces available	82	41.8	26	48.1
Disabled parking spaces available	10	5.1	6	11.1

⁎ *p* < .05.

While all websites mentioned a phone number, only two-thirds of websites specified the hours during which the dental practice can be reached by phone. Less than 25% of all websites mentioned sending appointment reminders, of which the majority concerned a notification by e-mail.

The majority of websites mentioned a phone number in case of dental emergencies, including the hours during which this phone number can be reached (Table 2). However, only 2 practices (2,5%) in the sample group offered emergency oral healthcare at the same location as the general oral healthcare. For all other practices, the website either indicated emergency care locations elsewhere or did not clearly specify.

Information on accessibility was limited; less than half of the websites mentioned availability of regular parking spaces, and only a few websites mentioned the availability of disabled parking spaces.

### Composition of the dental practice team

Table 3 shows the composition of the dental team as presented on the websites. Unsurprisingly, websites of the gero-group significantly more often mentioned the presence of a dentist with a specialisation in gerodontology compared to websites of the sample-group. Regarding the other categories, no differences were found between both groups. Overall, the majority of websites mentioned the presence of professionals aimed at prevention (i.e., dental hygienists and prevention assistants), while orthodontists, oral maxillofacial surgeons and non-dental professionals (category ‘other’) were seldom present. Very few websites were unclear about the composition of the dental practice team.

**Table 3:** Professionals within the dental practice team, as mentioned on the website, per group

	Sample-group (n = 196)		Gero-group (n = 54)	
	n	%	n	%
General dentist	185	94.4	49	90.7
Dental hygienist	149	76.0	34	63.0
Prevention assistant*	137	69.9	35	64.8
Practice manager	90	45.9	21	38.9
Specialised dentist (other than gerodontology)	70	35.7	15	27.7
Dental technician	47	24.0	11	20.4
Orthodontist	7	3.6	2	3.7
Oral maxillofacial surgeon	6	3.1	-	-
Specialised dentist in gerodontology‡	1	0.5	6	11.1
Other†	3	1.5	1	1.9
Unclear/not mentioned	9	4.6	3	5.6

⁎ Dental assistant with further education in prevention and oral hygiene.

† Anesthesiologist, physiotherapist, psychologist.

‡ *p* < .05.

### Information for elderly patients

Results on information aimed at elderly patients are presented in Table 4. Websites in the gero-group significantly more often emphasised the elderly as a specific target patient group compared to the sample-group (11,1% vs 2,6%). However, numbers were low overall. Additionally, only one website in the gero-group, and none in the sample-group, mentioned the possibility of a consultation concerning salivation problems.

**Table 4:** Information aimed at elderly patients mentioned on the website, per group

	Sample-group (n = 196)		Gero-group (n = 54)	
	n	%	N	%
Elderly mentioned as specific patient group§	5	2.6	6	11.1
Possibility of a consultation concerning salvation problems	-	-	1	1.9
Possibility of a home visit	1	0.5	2	3.7
Support for disabled people and the elderly mentioned §	38	19.4	23	42.6
- Wheelchair accessibility §	*34*	*17.3*	*20*	*37.0*
- Elevator	*2*	*1.0*	*3*	*5.6*
- Accessible toilet	*2*	*1.0*	*1*	*1.9*
- Patient lift (patient hoist)	*1*	*0.5*	*3*	*5.6*
- Automated external defibrillator (AED)	*1*	*0.5*	*2*	*3.7*
- Chair lift	*1*	*0.5*	*-*	*-*
- Wheelchair tilter	*-*	*-*	*1*	*1.9*
- Kiss & Ride spot	*-*	*-*	*1*	*1.9*
- Automatic (sliding) doors	*-*	*-*	*-*	*-*
- Supporting handles	*-*	*-*	*-*	*-*
- Movable dental unit	*-*	*-*	*-*	*-*
Clickable link to the website of Ivoren Kruis*	95	48.5	28	51.9
KIMO CPGs mentioned†	1	0.5	-	-
Referral to website of *De Mond Niet Vergeten*‡	2	1.0	-	-

⁎ “Ivory Cross”; the Dutch association for prevention and oral health.

† *Kennis Instituut Mondzorg (KIMO),* a Dutch non-profit foundation that develops clinical practice guidelines (CPGs).

‡ “Don't forget the mouth”; a Dutch initiative committed to improve community based oral healthcare for frail elderly people.

§ *p* < .05.

Only 3 websites offered the possibility of a home visit: one in the sample-group, providing treatment concerning removable dentures, and 2 in the gero-group, of which one offered regular check-ups and restorative treatments, while the other was unclear about the possibilities during a home visit.

Wheelchair accessibility was mentioned significantly more often in the gero-group compared to the sample-group (37,0% versus 17,3%), and 2 websites in the sample-group specifically mentioned that their dental practice is not accessible to wheelchairs. All other tools and features are rarely mentioned in both groups, and the vast majority of websites did not mention any at all.

Approximately half of the websites referred to the website of *Ivoren Kruis* (“Ivory Cross”; the Dutch association for prevention and oral health). However, almost none of them made a reference to the CPGs of KIMO or the website of *De Mond Niet Vergeten* (“Don't forget the mouth”; a Dutch initiative committed to improve community based oral healthcare for frail elderly people); only one website in the sample-group referred to the CPGs without mentioning a specific CPG, and 2 websites in the sample group referred to *De Mond Niet Vergeten*. Remarkably, none of the websites in the gero-group referred to either the CPGs nor *De Mond Niet Vergeten*.

## Discussion

The vast majority of Dutch dental practices seemed to have a website, with only 8% of the selected dental practices being excluded for not having one. Most websites mentioned opening hours, a phone number, and the composition of the dental practice team – information that could be relevant to all patients, including the elderly. However, regarding (frail) elderly patients as a specific patient group, information was overall found to be scarce. Surprisingly, websites of dental practices owned by or employing a dentist member of The Dutch Association for Gerodontology (gero-group) were hardly more informative or adjusted to the elderly than websites of a random sample of Dutch general dental practices. Only very few websites mentioned the elderly as a specific target patient group, even within the gero-group.

In general, there is plenty of room for improvement in website functionality. Although standard website templates commonly offer features that enhance functionality, the majority of the dental practice websites did not have any of the features that were screened for. Providing the possibility to adjust text size or read text aloud could aid elderly people with deteriorating vision and is therefore recommended for all websites.

Although opening hours were usually mentioned on the website, flexibility seemed scarce. Frail elderly patients are often dependent on working family members and/or informal caregivers for transportation. Thus, regular opening hours during the weekend or weekday evenings could be convenient to this patient group. However, this was not commonly offered, especially within the gero-group. Further, in case appointment reminders were sent, the majority were sent by e-mail. It would be interesting to investigate whether elderly patients prefer reminders by mail or phone, or whether it is preferred or possible to register the e-mail address of a family member and/or informal caregiver, as suggested by the practice guide of the Royal Dutch Dental Association.^6^

Information on emergency oral healthcare and the availability of (disabled) parking spaces was mostly missing. This information is essential for elderly patients with decreased mobility and/or dependence on others for transportation. For example, it would be convenient to know if emergency oral healthcare is provided in the same location as the general oral healthcare, instead of being outsourced to a dedicated dental emergency centre located further away – an increasing trend in the Netherlands, especially in urban areas. However, it is important to realise that the lack of information does not necessarily imply the absence of (disabled) parking spaces or emergency oral healthcare in the general practice. It mainly indicates the need for more detailed information on the websites.

Furthermore, apart from wheelchair accessibility, which was significantly more often mentioned in the gero-group, websites seldom mentioned the availability of tools and features that could support disabled people and the elderly. This emphasises the importance of improving information on websites, but possibly also indicates a lack of those tools and features within the general dental practice. However, tools like a chair lift or patient hoist can be expensive, and it may be unreasonable to expect general dental practices to have these specialised tools available, or to expect them to mention every possible tool on their website, including those not available. Future research could focus on identifying practices that are equipped with advanced tools, and then assess whether they properly report on these features on their website.

As hypothesised, very few websites mentioned the possibility of a home visit. Whenever possible, a visit to a dental practice is preferred due to the safe, controlled environment, proper equipment, and infection prevention measures. However, in (frail) elderly patients, physical disabilities and/or cognitive decline may complicate a visit to the dental practice, which may be a reason for a home visit according to the Dutch CPG on this topic. Although it is anticipated that home visits will become increasingly necessary due to the growing number of homebound elderly, it seems to be far from being a regular part of oral healthcare provided by general dental practices.

While the long-standing *Ivoren Kruis* (“Ivory Cross”; the Dutch association for prevention and oral health) seems to be commonly known and referred to by half of all websites, hardly any of the websites referred to either the CPGs of the more recently founded Dutch organisation for the development of CPGs (*Kennis Instituut Mondzorg*; KIMO) nor *De Mond Niet Vergeten* (“Don't forget the mouth”; a Dutch initiative committed to improve community based oral healthcare for frail elderly people), possibly indicating a lack of awareness of the existence of both. Especially for the gero-group, with an expected affinity for the elderly, this is a remarkable finding.

Altogether, the results of this study indicate room for improvement in website functionality and the information provided for (frail) elderly patients, as well as a need for improvement of the oral healthcare for this specific patient group. The results regarding website quality align with earlier studies that identified insufficient information on UK dental practice websites aimed at specific patient groups, for example, cancer patients,^9^ or specific treatment options, for example, aligner treatment^10^ and dental implants.^11^ An earlier study on Dutch websites of practices offering orthodontic treatment also concluded that readability of the information and website technical performance need further optimisation.^12^

However, an important limitation of the current study is, that the topics that were screened for were chosen by professionals, not patients. Although the practice guide of the Royal Dutch Dental Association, on which the screening instrument was based, is considered a valuable, carefully compiled and comprehensive document, no patients or patient group representatives were directly involved in the composition of the practice guide. We acknowledge the importance of including the (frail) elderly patients as a stakeholder group. Future research should therefore focus on mapping patient needs regarding dental practice websites, enabling tailored recommendations for improvement to match patient's needs. When providing information for (frail) elderly patients, it is also important to consider the needs of family members and/or informal caregivers, as they may search for information on behalf of the elderly. Furthermore, it is important to consider the quality and reading level of the information provided.^9^

In addition, research is needed to explore the importance of a website as a source of information for elderly patients in search of a dental practice. Literature on factors that influence a patient's decision when choosing a dentist is available, showing the importance of recommendations from the close social environment^13^, ^14^, ^15^ and qualifications and skills of the dentist,^13^^,^^14^^,^^16^^,^^17^ while the proximity of the dental office and the dentist's gender were considered less influential.^17^^,^^18^ With increasing age, wheelchair accessibility and dental office equipment become more important.^15^ Research on parents choosing a paediatric dentist for their child shows that most parents used Internet search engines and the practice website to obtain information.^14^ However, research on the importance of websites as an information source is scarce, especially for the elderly as a specific patient group, highlighting the need for further research in this area.

Further, it is common in the Netherlands to visit the dentist regularly for a dental check-up. However, not infrequently it turns out that elderly people no longer do this due to increasing frailty. In future research, it is therefore important to investigate how patients can remain involved in the dental practice they used to visit. This can be done, for example, by informing them about the importance of continued regular check-ups. This should not only be aimed at the elderly patients themselves, but also at their family members and/or informal caregivers. Future research could focus on the question of which forms of communication are preferred in this regard. More specifically, whether practice websites with extensive information about healthcare provision and accessibility can contribute to frail elderly people living at home continuing to visit a dentist regularly.

## Conclusion

Dental practice websites lack information in general, and the information provided relevant to elderly patients in particular. At the same time, there is room for improvement in website functionality. Practice owners could benefit from the development of further website guidance. To include desired information and optimal display methods in this guidance, future research should focus on mapping patient needs regarding dental practice websites. Additionally, general dental practices do not seem to be sufficiently equipped for the treatment of (frail) elderly patients, and seem to insufficiently adhere to currently available guidance on this topic, highlighting the need for improvement of oral healthcare for this specific patient group.

## Summary table

**Table:** 

What was already known on the topic - The number of elderly people and their need for dental care increases. - The vast majority of Dutch dental practices has a website; a possible valuable source of information for patients.
What this study added to our knowledge - Dutch dental practice websites lack information in general, and information relevant to elderly patients in particular. - General dental practices do not seem to be sufficiently equipped for the treatment of (frail) elderly patients.

## Author contributions

JMK contributed to the conceptualisation, methodology and analysis of the study, and drafted the manuscript. BYZL contributed to the methodology and data collection of the study, and reviewed and edited the manuscript. SJT contributed to the methodology and data collection of the study, and reviewed and edited the manuscript. JJMB contributed to the conceptualisation, methodology and analysis of the study, and reviewed and edited the manuscript.

## Funding

The collaboration project is co-funded by PPP Allowance awarded by Health-Holland, Top Sector Life Sciences & Health, to stimulate public-private partnerships.

## Conflict of interest

None disclosed.

## Appendix Supplementary materials

Image, application 1

Image, application 2

## Footnotes

Supplementary material associated with this article can be found in the online version at doi:10.1016/j.identj.2024.10.023.
