# Supplementary material for: Evaluation of the Information on Dutch Dental Practice Websites Regarding the Treatment of (Frail) Elderly Patients
Source: Int Dent J. 2024 Dec 16;75(2):1384–9. doi: 10.1016/j.identj.2024.10.023 (PMC11976547; doi:10.1016/j.identj.2024.10.023)
Supplement: Supplementary file 1 [file mmc1.docx]

**Evaluation of the information on Dutch dental practice websites regarding the treatment of (frail) elderly patients**

Johanna Margaretha Kroese; Brigitta Yue Zhi Li; Samuel Julian The; Jan Joseph Mathieu Bruers

**Appendix**

| **Website functionality** | | |
| --- | --- | --- |
| Which features are available that enhance functionality of the website? | | *zoom function / adjustment of text size / reading text aloud / search function / day and night mode / language change / none* |
| **Contact details and accessibility** | | |
| Are practice opening hours mentioned? | | *yes / no* |
| - | If yes, what are the opening hours? | *weekdays / weekday evenings / weekend / weekend evenings / appointment outside regular opening hours possible on request* |
| Is a phone number displayed? | | *yes / no* |
| - | If yes, is it mentioned during what hours the dental practice can be reached by phone? | *yes / no* |
| Is an emergency phone number displayed? | | *yes / no* |
| - | If yes, is it mentioned during what hours the emergency phone number should be used? | *yes / no* |
| - | Is emergency dental care provided at the same location as the general dental care? | *yes / no / unknown* |
| Is it mentioned that patients receive a reminder for their appointment? | | *yes / no* |
| If yes, using which method do patients receive a reminder for their appointment? | | *sms / e-mail / mail / by phone / unknown* |
| Are regular parking spaces available? | | *yes / no* |
| Are disabled parking spaces available? | | *yes / no* |
| **Composition of the dental practice team** | | |
| What professionals constitute the dental practice team? | | ** General dentist / dentist with a specialization in gerodontology / dentist with a specialization in a different area / dental hygienist / prevention assistant** / dental technician / orthodontist / oral maxillofacial surgeon / practice manager / other*** / unclear or not mentioned* |
| *  **  *** | The list of possible answers was supplemented during collection of the data based on findings, resulting in a list of 41 possible categories. For data analyses, these were clustered in the 11 categories listed in this table.  Dental assistant with further education in prevention and oral hygiene  anesthesiologist, physiotherapist, psychologist | |
| **Information for elderly patients** | | |
| Are elderly mentioned as specific target patient group? | | *yes / no* |
| Is the possibility of a home visit mentioned? | | *yes / no* |
| - | If yes, which treatments are offered during a home visit? | *regular check-up / treatment concerning removable dentures / dental cleaning / restorative treatment / tooth extractions / root canal treatments / other, … / unclear* |
| Is the possibility of a consultation concerning salivation problems mentioned? | | *yes / no* |
| What tools or features are mentioned to support disabled people and the elderly? | | *Wheelchair accessibility / wheelchair inaccessibility / automatic (sliding) doors / supporting handles / accessible toilet / elevator / chair lift / patient lift (patient hoist) / automated external defibrillator (AED) / movable dental unit / wheelchair tilter / Kiss & Ride spot / none mentioned* |
| Are clinical practice guidelines (CPGs) of the Dutch organization for the development of CPGs (*Kennis Instituut Mondzorg*; KIMO) mentioned? | | *yes / no* |
| - | If yes, which clinical practice guidelines are mentioned? | *Dental care for home bound frail elderly / Root caries in elderly / Xerostomia and hyposalivation related to medication and polypharmacy / Overdenture on implants in the edentulous maxilla / Overdenture on implants in the edentulous mandible / other/not specified* |
| Is there a clickable link to the website of *Ivoren Kruis* (“Ivory Cross”; the Dutch association for prevention and oral health)? | | *yes / no* |
| Is there a referral to the website of *De mond niet vergeten* (“Don’t forget the mouth”; a Dutch initiative committed to improve community based oral healthcare for frail elderly people)? | | *yes / no* |

**Appendix Table 1. Items on the registration form used to screen dental practice websites on information relevant to elderly living at home and seeking dental care.**
